# MINDDS-connect: a federated data platform integrating biobanks for meta cohort building and analysis

**DOI:** 10.1038/s41431-025-01927-5

**Published:** 2025-08-20

**Authors:** Benjamin Huremagic, Nishkala Sattanathan, Mathilde Geysens, Janet Harwood, Jente Verbesselt, Senne Meynants, Ann Swillen, Kris Van Den Bogaert, Danijela Drakulic, Goran Cuturilo, Thérèse van Amelsvoort, David Linden, Natália Oliva-Teles, Paula Jorge, Marianne B. M. van den Bree, Jessica H. Hall, Haleh Chizari, Amin Ardeshirdavani, Adrian J. Harwood, Geert Vandeweyer, Yves Moreau, Joris Robert Vermeesch

**Affiliations:** 1 Laboratory of Cytogenetics and Genome Research, Centre for Human Genetics, KU Leuven, Leuven, Belgium; 2 Department of Medical Genetics, University of Antwerp, Antwerp, Belgium; 3 Department of Human Genetics, Centre for Human Genetics, University Hospitals Leuven, Leuven, Belgium; 4 Neuroscience and Mental Health Innovation Institute (NMHII), Cardiff University, Cardff, UK; 5 School of Biosciences, Cardiff University, Cardiff, UK; 6 Laboratory for Behavior and Neurodevelopment, Centre for Human Genetics, KU Leuven, Leuven, Belgium; 7 Institute of Molecular Genetics and Genetic Engineering, University of Belgrade, Belgrade, Serbia; 8 Faculty of Medicine, University of Belgrade, Belgrade, Serbia; 9 University Children’s Hospital, Belgrade, Serbia; 10 Mental Health and Neuroscience Institute (MHeNs), Maastricht University, Maastricht, The Netherlands; 11 Unidade Local de Saúde de Santo António, Centro Hospitalar Universitário de Santo António, Porto, Portugal; 12 UMIB-Unidade Multidisciplinar de Investigação Biomédica, ICBAS-Instituto de Ciências Biomédicas Abel Salazar, Universidade do Porto, Porto, Portugal; 13 ITR-Laboratory for Integrative& Translational Research in Population Health, Porto, Portugal; 14 MEDCIDS - Departamento Medicina da Comunidade, Informação e Decisão em Saúde, Faculty of Medicine, University of Porto, Porto, Portugal; 15 Centre for Neuropsychiatric Genetics and Genomics, Division of Psychological Medicine and Clinical Neurosciences, Cardiff University, Cardiff, UK; 16 ESAT-STADIUS, KU Leuven, Leuven, Belgium; 17 Department of Medical Genetics, University Hospital of Antwerp, Edegem, Belgium

**Keywords:** Neurodevelopmental disorders, Medical research, Databases

## Abstract

Access to large patient cohort data and biobanked resources is a catalyst for progress in genomics and biomedical research, increasing statistical power, and unlocking deeper insights—especially in areas like rare diseases and mental health. Responsible research necessitates maintenance of data privacy, regulatory compliance, and research standardization. It can appear that these guiding principles oppose each other and present barriers to responsible Open science. To address these critical challenges, we developed MINDDS-Connect, a federated data collaboration platform that integrates a web-based interface with decentralized Docker instances via a REST API. This architecture allows registered users to securely query samples across the platform’s network, and offers a tool to facilitate the formation of virtual multi-centric meta-cohorts and research collaboration. MINDDS-Connect allows institutions to retain data control while enabling collaborative research and meta-cohort analysis through standardized metadata fields. Its implementation across five European centers enhanced the accessibility of 900 samples, demonstrating its effectiveness in enabling cohort construction and promoting collaborative research. The platform provides a secure, open-source solution consistent with EU Open Science policies, advancing large-scale mental health research.

## Introduction

Access to biological and clinical data is a foundational pillar within biomedical sciences. It is the driver for research collaboration and instrumental for the accumulation of our collective research knowledge. There are strong medical innovation, financial, and even ethical benefits from maximizing the usage of patient data and resources collected at multiple sites to create meta-cohorts. This is particularly relevant for rare disorders (RDs) given their low frequency in the general population and geographical dispersion. Analysis of meta-cohorts will speed up gene discovery and ultimately lead to improved therapeutic strategies, identify targets for drug development, inform health policies, and drive a new era of Precision Medicine.

However, a significant roadblock for RD research is a perceived challenge to balance access to patient data and biological samples against the fundamental rights of individuals to the privacy and data security. On one side, FAIR (Findable, Accessible, Interoperable, Reusable) principles promote better use of patient-based research data, and also by encouraging standardized formats and informative metadata. On the other, the rights of the individual must remain unassailable, and data protection is a significant economic and security issue. Stringent data protection regulations, such as the General Data Protection Regulation (GDPR), often pose a significant obstacle for data sharing [1]. While necessary, these regulations introduce non-trivial consent and data management requirements, which may deter researchers from collaborating. Additionally, lack of data standardization complicates the integration process due to differences in data quality, format, and accessibility [2]. These issues are enhanced for rare disorders, where the uniqueness of an individual’s clinical profile may unintentionally lead to de-anonymization.

Advanced sequencing technologies and the detection of genome variants (single nucleotide variants (SNVs) and copy number variants (CNVs)) allow us to study patients with a shared genetic etiology, thus employing a “genotype-first approach” rather than the classical “phenotype-first approach” traditionally used in psychiatry [3]. However, interpreting genomic data highly depends on the availability of access to databases containing genetic variants from both control population and other patient cohorts. Considering the heterogeneity of genetic disorders, in-house data, used for variant filtering, is often insufficient to provide an accurate molecular diagnosis. A survey conducted by the ‘Maximising impact of research in neurodevelopmental disorders’ (MINDDS) consortium across 30 European centers identified more than 3800 carriers of CNVs related to neurodevelopmental disorders (NDDs) [4]. This result showed that precise data-sharing approaches would benefit clinical and research purposes.

In response to these challenges, we present MINDDS-Connect, a federated data-collaboration platform. In contrast to traditional centralized databases, MINDDS-Connect offers a solution that retains data within the institutions of origin. This federated approach complies with GDPR requirements, addressing critical data privacy issues [5]. Additionally, it provides for data standardization, facilitating accessibility and utilization of data in a consistent and efficient way. By compiling of only high level metadata for data and biological samples, such as age at sampling, sex, stored material type with Human Phenotype Ontology (HPO) terms [6], and Online Mendelian Inheritance in Man (OMIM) [7], it offers discoverability, whilst maintaining data privacy. Thus MINDDS-Connect enables researchers to select the most appropriate data and samples for their studies based on simple, high level criteria. The platform allows for the categorization of samples into public or private categories, giving the local data custodians complete control over data.

As a proof of concept, we focused on individuals with NDD, including intellectual disability (ID), Autism Spectrum Disorders (ASD), with or without congenital malformations [8–10]. These disorders significantly contribute to the population’s mental health burden [11]. Importantly, the strong correlation between specific gene loci and (neuro) developmental risk creates an opportunity to establish the mechanisms underlying the risk of transitioning from health to illness. However, the scarcity of high-risk individuals in the overall NDD patient population would greatly benefit from data aggregation and identification of sample type for cohort building and meta-analysis [12]. Five centers collaborated (Table 1) within the MINDDS-Connect network to create a 900 sample prototype, and demonstrate a use case based on the 22q11.2 CNV. MINDDS-Connect represents a step forward in addressing the challenges of RD research. By providing a secure, flexible, and standardized platform for data sharing, it empowers researchers to collaborate. As the network expands and more data is included, MINDDS-Connect has the potential to revolutionize RD research and improve patient’s care.

**Table 1:** Sample contributions by the five centers connected within the MiNDDS-Connect Network.

Centers	Shared Samples
UZ Leuven/KU Leuven	413
Cardiff University	220
IMGGE Belgrade	26
Maastricht University	241
Centro Hospitalar Universitário de Santo António	0
	900

The table shows the number of shared samples contributed by each center.

## Materials and Methods

**Fig. 1:**
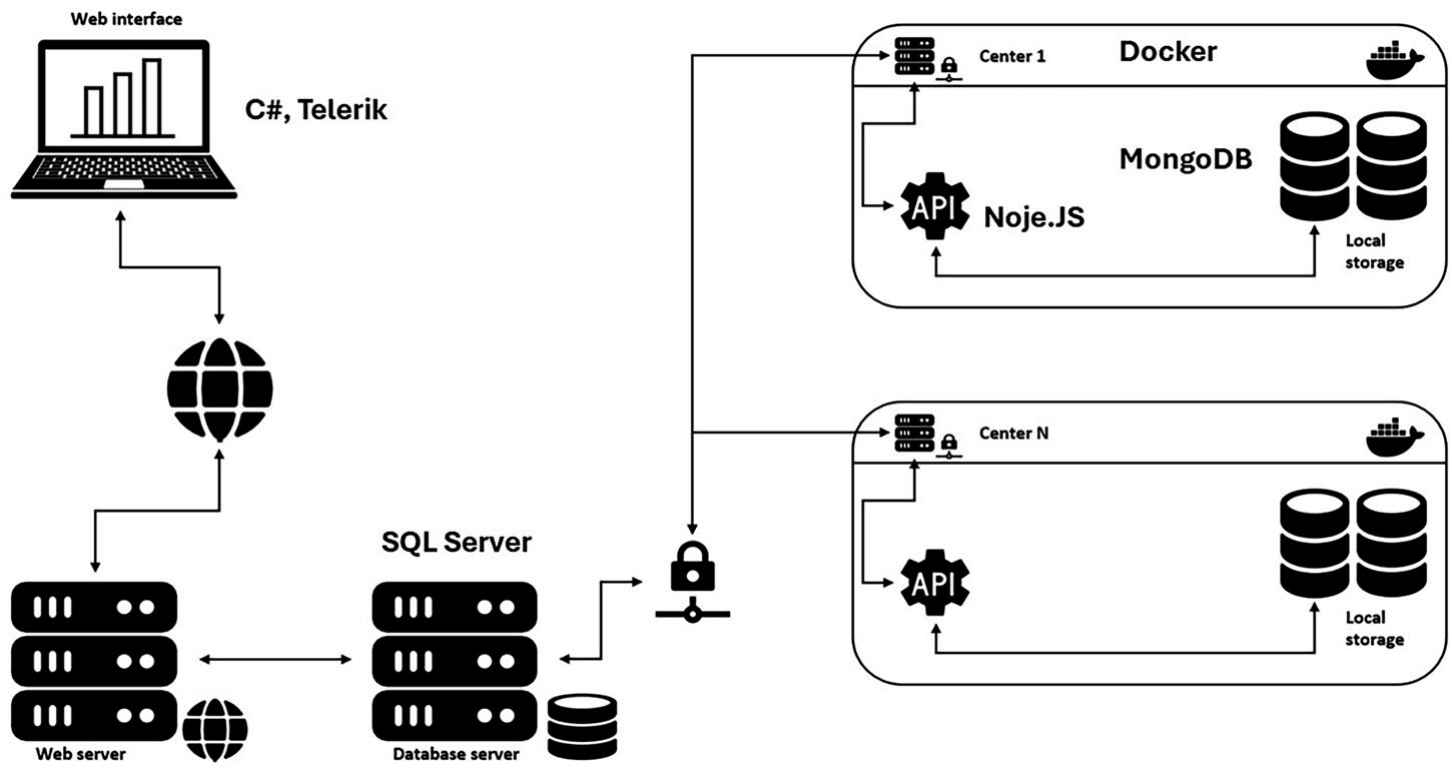
MINDS-Connect IT architecture. Schematic overview of the IT structure.

MINDDS-Connect comprises four main components: a user interface (UI), a central database, a decentralized database, and a Representational State Transfer Application Programming Interface (REST API). The UI is developed using C# (ASP.NET), Telerik framework, and JavaScript. The central database that manages the access privileges through the access control list (ACL) is developed using Microsoft SQL Server. The decentralized database relies on a NoSQL database, MongoDB, and stores data within the client of origin. The REST APIs are developed using Node.js to enable communication between the central database and network clients. However, the NoSQL database and REST APIs are encapsulated within a Docker to simplify the installation procedures and solve potential software compatibility issues. (Fig. 1).

After installing the Docker image, every new client that joins the network must provide the IP and URL for client APIs and the authentication credentials. After this, the Local Administrator (LA) and first Principal Investigator (PI) are registered for the client, and the client is set online inside the MINDDS-Connect network. Other users linked to the client can then register, with their admission managed by the local admin. Upon logging into the UI, users automatically get authenticated in the client of origin.

### Access Control List (ACL)

**Fig. 2:**
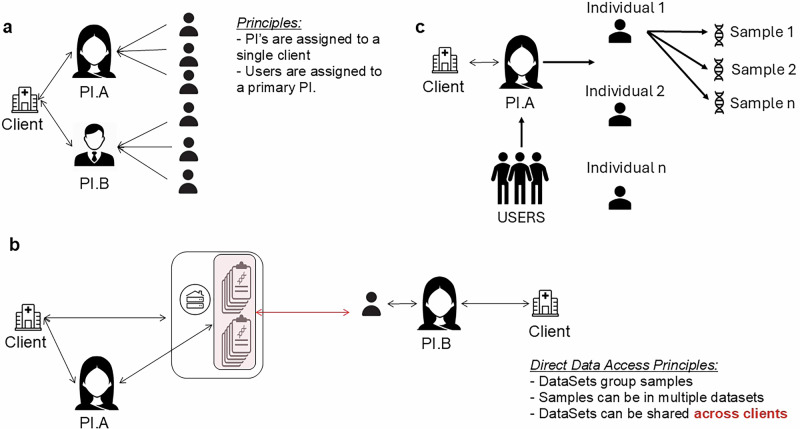
MINDDS-Connect structure. **a** User structure, **b** direct data access across centers through datasets, **c** Data structure.

The access control list (ACL) represents the platform’s core and it enables complete management of data. Through ACL, access can be regulated at intra-client and inter-client levels. At a client level, there are three user types: Local Administrator (LA), Principal Investigator (PI), and User. The LA’s responsibility is to manage user registration. PIs are the custodians of the data, and all the samples inserted are linked to a PI (Fig. 2a, c). Ultimately, users represent the associates of the PI and are granted access and allowed to input data for the PI. To enable intra-site collaboration, LAs can link users to multiple PIs within the client. Alternatively, users can be assigned to user groups to which private data can be exposed. To enhance collaboration at the inter-client level, the ACL allows organization of data into datasets that can be shared with users from different clients (Fig. 2b).

### Data structure and data standardization

Data is structured into individuals and samples to guarantee consistency. Samples can be associated only with one individual, while every individual can have multiple samples. Individuals can be described using a Local Individual ID, sex, and HPO terms [10]. Subsequently, samples can be described with additional metadata: age at sampling, stored material type, anatomical site, diagnosis type, availability of genomic data, and OMIM terms. Samples can be private or public and consequently findable by other platform users. To ensure data standardization across different centers, all fields offer a list of values that users can select from when inputting data. Ultimately, data input can be managed in two ways: through the UI, where users can insert one sample at a time, or through a structured CSV file in case of a bulk upload. When preparing the CSV file, users must adhere to the provided encoding (Supp. File 1) as shown in example (Supp. File 2). Entries that do not comply with the encoding format will be rejected. Upon upload, data is stored within the institute of origin, while the central database stores system-generated IDs that allow data retrieval for visualization in UI.

### User interface of MINDDS-connect

#### “MySamples”

The “My Samples” provides access to the samples uploaded by a PI’s group, and displays an overview of managed samples with the following structure:

*Table Layout*: samples are organized in a tabular format, and each row represents a unique sample. Columns provide information about the sample and individual through following attributes “Individual ID,” “Local Individual ID,” “Sample ID,” “Local Sample ID,” “Sex,” “Age at Sampling,” “Material,” “Anatomical Site,” “Diagnosis,” “OMIM ID,” “HPO,” “Genomic Data,” “Center Description,” “PI Name,” and “Access.”

*Filtering*: Samples filtering can be performed for specific sample criteria or IDs by using the filtering icon on column headings.

*Sample Insertion*: Upload multiple samples via CSV (“Upload Selected File”), or add a single sample via “Add New Sample” button. *Downloading Samples*: A list of samples can be downloaded through “Download Samples” button for offline analysis or record-keeping.

The “My Samples” represents the data management dashboard. PIs and associated researchers can update the sample information and change the sample’s visibility from private to public within the platform.

#### “Catalog”

The “Catalog” displays the metadata of samples shared by other centers within the platform. Similarly to “My Samples,” samples are organized in a tabular format. However, “Catalog” does not include the local identifier columns for the individuals and samples. Information about the PI responsible for the sample can be retrieved through the “PI Name” column. The main purpose of the “Catalog” section is to enable users to search for visible samples across different centers. The cross-center search functionality enhances data discoverability and facilitates collaborative research efforts.

The platform presents additional UI sections that are visible exclusively to PIs and LAs. (Supp. File 3).

## Results

**Fig. 3:**
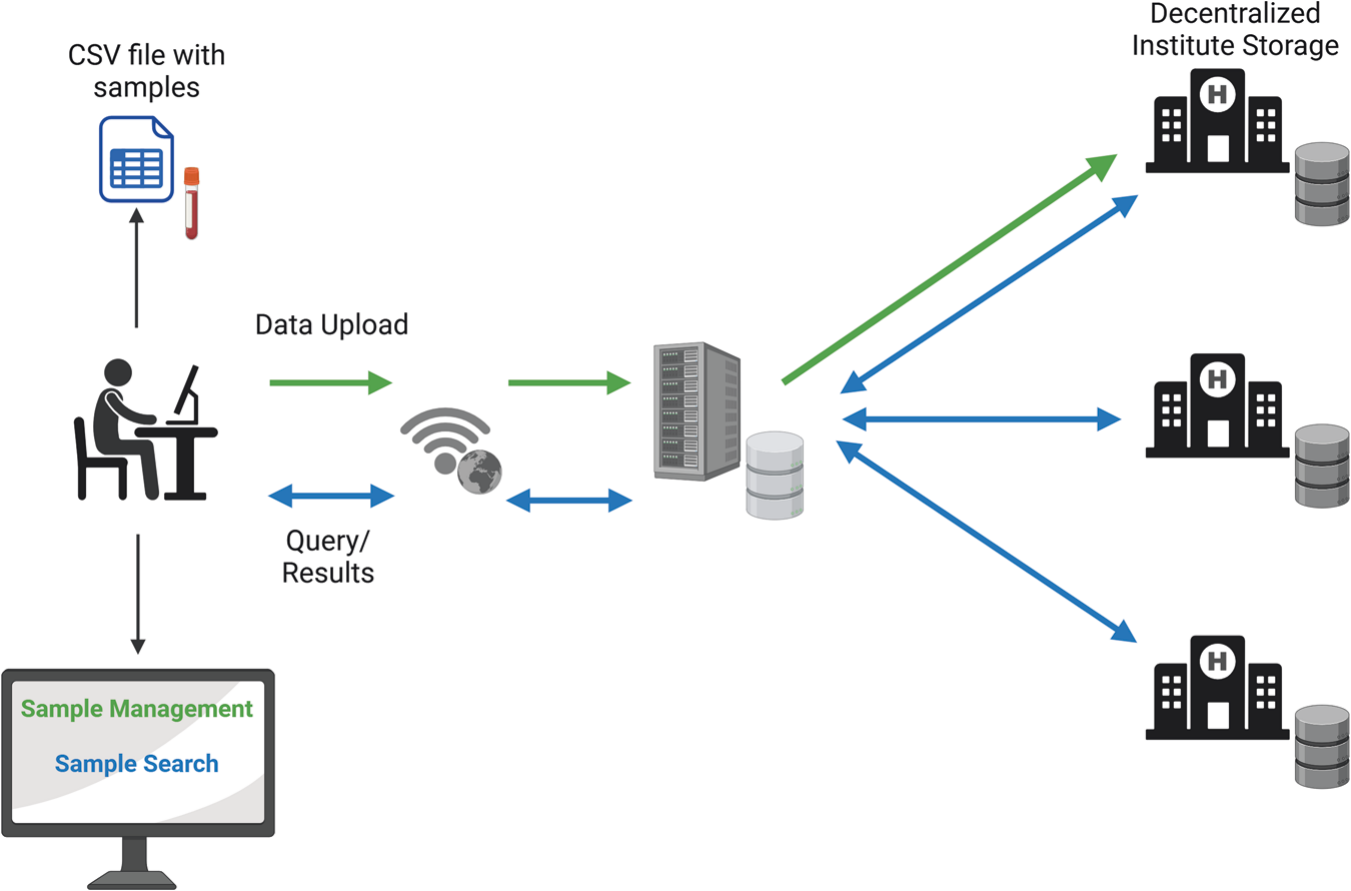
Fundamental functionalities of the platform. Overview of the platform usage from users perspective, from data upload to data discovery.

The UI of MINDDS-Connect offers a standardized approach for sharing and managing the availability of biological samples. The platform functions as a local sample database and allows users to manage their samples and search for samples within the platform. (Fig. 3).

In piloting the MINDDS-Connect platform, five centers were connected to demonstrate the system’s functionalities and data-integration capabilities. An overview of all the connected centers, PIs and samples in the platform is shown in Table 1.

### Searchable samples – identifying most frequent disorder

The MINDDS-Connect platform was designed to facilitate sample discovery and cohort meta-analysis, in neurodevelopmental disorder research and foster collaborative opportunities by providing researchers with access to a diverse and well-curated sample catalog. To demonstrate the potential of this resource, five sites provided data on patients carrying rare CNVs, SNVs or without a diagnosis. 900 samples were entered. Initially, we stratified the 900 samples through the platform’s “Catalog” and identified 36 control samples, 277 patients without a known pathogenic variant following whole genome sequencing, and 587 samples associated with an OMIM ID (Table 2).

**Table 2:** Distribution of 900 shared samples in the MINDDS-Connect Network by diagnostic category and OMIM.

Shared Samples	Count
**Samples with OMIM ID**	**587**
*- OMIM:611867 (22q11.2 DEL)*	*269*
*- OMIM:608363 (22q11.2 DUP)*	*102*
*- OMIM:611913 (16p11.2 DEL)*	*121*
*- OMIM:614671 (16p11.2 DUP)*	*61*
*- Other*	*34*
**Diagnosis Not Specified**	**277**
**Controls**	**36**
**Total**	**900**

The majority (587 samples) are linked to an OMIM, including 22q11.2 deletions (269) and duplications (102), and 16p11.2 deletions (121) and duplications (61). An additional 277 samples have unspecified diagnoses, and 36 are controls, highlighting the dataset's focus on 22q11.2 and 16p11.2 CNVs.

To identify the most frequent disorders within the cohort we focused on the 587 OMIM-associated samples. Since participating sites are involved in NDD research, a significant proportion of samples (371) showed abnormalities in the 22q11.2 chromosomal region, encompassing both Deletion (269) OMIM:611867 and Duplication (102) OMIM:608363 Syndromes. The 16p11.2 region, associated with Deletion (102), OMIM:611913, and Duplication (61), OMIM:614671, syndromes, was the second most frequently represented region with 182 samples. Additionally, 34 samples were associated with other rare genetic disorders (Table 2).

### Phenotypic characterization of neuropsychiatric outcomes in 22q11.2 CNVs

With 22q11.2 deletions and duplications being the most represented CNVs as well as one of the most common causes of NDDs, we set out to analyze these samples based on their phenotype. 22q11.2 deletion syndrome (22q11.2DS) patients have a high risk of developing neuropsychiatric phenotypes. Anxiety is well-documented as a precursor to psychosis in individuals with 22q11.2 deletions and duplications, often acting as a trigger during adolescence [13, 14]. We first explored the incidence of anxiety disorder (HP:0000739). From the original 371 patients, 55(20.4%) deletion carriers - and 30(29.4%) duplication carriers had a diagnosis of anxiety disorder, with no statistically significant difference observed between deletions and duplications (*p* = 0.07). Notably, 47 of them were under the age of 18, comprising 30 deletion and 17 duplication carriers. Autism Spectrum Disorder (ASD) (HP:0000717) is also commonly associated with both 22q11.2 deletion and duplication syndromes [15]. When filtering for ASD, we observed 74(27,5%) cases among deletion carriers and 26(25,4%) among duplication carriers, with no statistically significant difference observed between the rearrangements (*p* = 0.78).

**Fig. 4:**
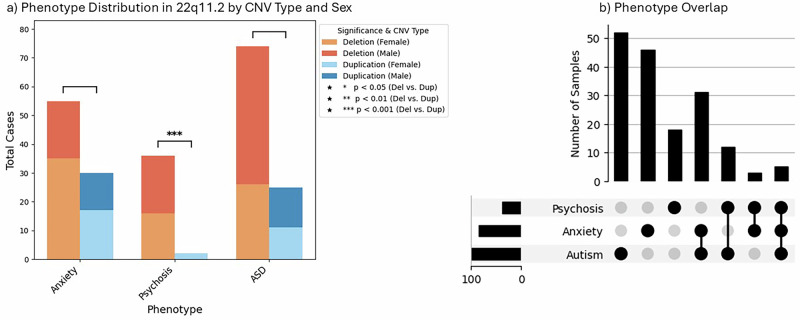
Use case exploring Phenotype Distribution in 22q11 by CNV Type. **a** Phenotype Distribution by CNV Type and Sex: This stacked bar plot represents the distribution of three psychiatric phenotypes (Anxiety, Psychosis, and Autism Spectrum Disorder (ASD)) among individuals with 22q11.2 deletions and duplications, stratified by sex. The bars are color-coded to distinguish between deletion (female and male) and duplication (female and male) cases. Statistical significance for deletion versus duplication comparisons is indicated by brackets and asterisks (*p < 0.05, **p < 0.01, ***p < 0.001), with psychosis showing significance. **b** Phenotype Overlap Among Affected Individuals: UpSet plot illustrates the occurrence of psychosis, anxiety, and autism, and their overlaps.

22q11.2DS is strongly associated with psychosis and schizophrenia. Duplications, on the other hand, are often associated with milder phenotypes or isolated traits [16, 17]. We filtered the entire 22q11.2 CNV subset for the psychosis phenotype (HP:0000709), identifying 38 individuals. Among them, 36 (13.3%)carried a deletion, while only 2(0.01%) carried a duplication, indicating a significantly higher incidence of psychosis in deletion carriers (*p* = 0.0004). Psychosis was predominantly observed in adults, with just 3 affected individuals (7.9%) under the age of 18. This age distribution aligns with existing literature suggesting that psychosis in 22q11.2DS commonly manifests during late adolescence or early adulthood, a critical period for brain maturation and heightened stress vulnerability [13, 18]. (Fig. 4a, b, Sup. File 4).

Additionally, we examined the sex distribution in the 22q11.2 cohort. The cohort consisted of 162 males and 206 females including 113 males and 156 females 22q11.2DS. Although there was a slightly higher representation of females among deletion carriers, the relative proportions of males and females allowed for further analysis of sex-specific differences in neuropsychiatric phenotypes.

Of the 55 individuals with a deletion and anxiety, 35 were female, and 20 were male. The frequency of anxiety was comparable between males (17.7%) and females (22.4%), with no statistically significant difference observed (*p* = 0.36). For psychosis, which was identified in 36 deletion carriers, 20 were male (17.7%) and 16 were female (10.3%), reflecting a slightly higher frequency of psychosis in males, however, this difference was not statistically significant (*p* = 0.10). Among the 74 deletion carriers with ASD, 48 were male (42.5%) and 26 were female (16.7%), indicating a significantly higher incidence of ASD in males (*p* < 0.00001) (Fig. 4a).

These findings highlight sex-specific differences in the phenotypic presentation of 22q11.2DS. While anxiety showed similar proportions between males and females, psychosis was slightly more prevalent in males, and ASD was significantly more frequent in males. Studies have reported a male-to-female ratio of approximately 2.3:1 in ASD among individuals with 22q11.2DS [19]. Additionally, while the prevalence of ASD in the general population follows a 3:1 male-to-female ratio, the pattern persists among individuals with 22q11.2DS [20]. These observations indicate that males exhibit a higher susceptibility to more severe neuropsychiatric phenotypes, aligning with previous research.

### Exploration of sample availability to engage in collaborative research projects

The platform allows researchers to explore the presence of patients, patient records, DNA, cell lines, the cell line types (Epstein Barr transformed B lymphocytes, fibroblasts, induced Pluripotent Stem Cells(iPSCs), …) across centers. Through the platform’s search features, investigators can determine whether the necessary samples and data are available to support their proposed research. This functionality enables planning collaborative or multi-center studies, where access to diverse samples is essential for feasibility and success. Researchers can enhance their projects’ competitiveness and potential impact by utilizing the platform in the grant drafting stage. The 269 22q11.2 deletion and 102 duplication derived samples were spanning across four sites. To further explore the feasibility of collaborative research, we analyzed the material types available for these 371 samples. For 93 samples DNA was available, for 6 iPSCs, 1 had neural progenitor cells, while the remaining 271 lacked specific material information or had unknown material types. This detailed stratification of material types highlights the platform’s utility in facilitating collaborative projects. For instance, researchers interested in 22q11.2DS could use this information to establish cohorts and further collaborations.

## Discussion

NDDs are heterogeneous and individually rare genetic conditions, necessitating collaborative research to understand their cause, improve therapies and establish predictive measures for early diagnoses. Collaborative approaches based on meta-cohorts can increase sample sizes in a cost-effective way. MINDDS-Connect aims to deliver a streamlined and ethically sound data platform for research collaboration. It addresses critical challenges in genetics and mental health research by providing a secure,standardized framework for integration and analysis of sensitive patient data to advance multi-site collaboration. It will facilitate data findability and accessibility as well as standardized metadata across geographically distinct research centers, delivering for EU Open Science policies and FAIR principles.

Most sample catalogs are siloed within institutions and inaccessible to external scientists. To collaborate, commonly data and bioresource sharing relies on depositing data in centralized repositories. While solutions like RD-Connect’s Sample Catalog [21], are beneficial for data integration, they often face challenges related to data privacy, and governance/ownership [22]. Federated data approaches allow data to remain within institutional boundaries while making metadata accessible for research purposes. This approach supports compliance with privacy regulations such as GDPR and helps to address concerns about data access and regulation. Although federated platforms offer advantages, implementation barriers remain. Implementing federated platforms can be complex, requiring robust infrastructure and IT expertise. These approaches are resource-intensive, demanding consistent data harmonization and interoperable data standards, which can be difficult to achieve across different institutions. Additionally, federated platforms rely on strong governance frameworks to manage data access and ensure security, adding to the complexity of their implementation.

Despite challenges, federated approaches are becoming established in the biomedical and healthcare sectors as they more easily accommodate the sensitive nature of health-related data, simplify the protection of patient privacy and ensure compliance with regulations such as GDPR and HIPAA [23]. These approaches improve healthcare delivery by integrating data from diverse sources, including electronic health records (EHRs), genomic databases, and biobanks. For instance, the German Biobank Alliance adopts a federated infrastructure based on HL7 FHIR standards to provide access to biosample metadata across multiple sites, enhancing data accessibility while mitigating privacy concerns [24]. Similarly, the Global Alliance for Genomics and Health (GA4GH) Beacon Project [25, 26] offers a federated framework that enables researchers to query genomic datasets across distributed nodes while maintaining local control and regulatory compliance.

However, these platforms often lack support for RD research, where deep phenotyping is critical. MiNDDS-Connect uniquely emphasizes metadata-first sample discovery using HPO and OMIM standards, which are crucial for harmonized case matching in rare neurodevelopmental disorders. Additionally, MiNDDS-Connect is lightweight to deploy via Docker and includes role-based access controls, making it suitable for smaller institutions.

Existing federated solutions perform well but present challenges for RDs. The lack of granular phenotyping method complicates the accurate characterization of individuals with rare conditions, creating a gap that MiNDDS-Connect seeks to address through standardized, metadata-driven matching. (Table 3).

**Table 3:** Comparative Overview of Data Discovery Platforms for Rare Disease Research(.xlsx): Side-by-side comparison of four data discovery platforms across five key criteria: metadata architecture, ontology integration, deployment, data model, and overall rare-disease suitability.

Platform	Metadata-first	HPO/OMIM support	Dockerized Deployment	Federated Model	Rare Disease Suitability
MiNDDS-Connect	Yes	Yes	Yes	Yes (metadata federated, local control)	High (deep phenotyping + metadata search)
GA4GH Beacon v2	No	No	No	Yes (query federation, local control)	Medium (genomic presence/absence queries)
German Biobank Alliance	Partial	No	No	Yes (FHIR-based, decentralized access)	Medium (biosample access, limited phenotype detail)
RD-Connect Sample Catalog	Partial	No	No	No (centralized repository)	High (centralized data, lacks standardization)

The federated platform described here employs a central hub to enable users to securely access their specific decentralized instances. This system follows a federated governance framework, where the central hub coordinates metadata and access protocols while the actual data remains decentralized and under the control of its original custodians [27].

This framework is particularly suited to managing sensitive patient data, which must remain localized. It offers a scalable and secure framework for distributed environments, ensuring both accessibility and compliance. In addition we have placed a strong emphasis on standardizing phenotypic information using HPO terms and OMIM identifiers, to address the need to characterize individual with rare conditions. HPO terms provide a standardized vocabulary for phenotypic diagnoses and symptoms, enabling consistent and precise phenotyping across different datasets, which is crucial for accurate disease characterization and cross-study comparisons [6].

### MINDDS-connect: enhancing data standardization and promoting collaborative research

Data standardization across different research centers is essential to maximize data interpretability and facilitate meaningful meta-analyses. MINDDS-Connect structures data into standardized phenotypic and biological samples from participants. By using HPOs, researchers can effectively capture and communicate nuances of phenotypic features. This is especially important in complex or rare conditions where precise terminology is critical for accurate diagnosis and data integration [6]. OMIM serves as the gold standard for genetic disorder information, providing a comprehensive catalog of human genes and genetic phenotypes, essential in ensuring accurate and consistent genotype-phenotype correlations. [7]. This standardization enhances the searchability, integration, and comparison of data from various sources, improving research reliability and enabling large-scale studies.

The platform’s UI and ACL provide a framework for managing data access and visibility, enabling researchers to search for and access samples across multiple centers. This capability enhances collaboration by supporting use cases critical to neurodevelopmental disorder research, including cohort construction, sample sharing, reanalysis of undiagnosed samples, and exploration of sample availability for proposal development.

### Strengths and weaknesses of MINDDS-connect

The MINDDS-Connect federated model ensures compliance with GDPR and allows for privacy-preserving data access. The use of controlled vocabularies (OMIM and HPO) for data annotation enhances interoperability and quality of the data. This data standardization is significant strength compared with other platforms. While MINDDS-Connect enables federated metadata discovery, all actual data or sample sharing occurs outside the platform, under the discretion of participating centers. Each institution is responsible for ensuring Institutional Review Board (IRB) or ethics committee approval before making metadata discoverable.

However, the initial IT requirements to set up a federated platform, could present a barrier for smaller institutions with limited resources. To address this challenge, we have implemented a streamlined installation process using Dockerization, which simplifies deployment and reduces the technical expertise required for setup.

#### Scalability and roadmap for Federated analysis

MINDDS-Connect currently supports metadata-level queries, but is designed as a part of federated ecosystem WiNGS (Widely Integrated NGS Platform) designed to enable more advanced federated analytics. This integration allows users registered on both platforms to share biological samples through MINDDS and genomic data through WiNGS [28]. This will enable summary statistics and customizable genomic queries for SNVs and SVs, with privacy-preserving computation enabled via secure multiparty computation: each decentralized instance processes data locally and shares only summarized outputs for aggregation. All the modules are implemented using modular Dockerized services interacting with the core MongoDB and API layers. To improve scalability, the central hub is being adapted for dynamic cloud deployment to accommodate higher loads. To prevent overloading decentralized nodes, API call frequency will be managed via rate-limiting, ensuring stable performance even for more complex queries and high query volumes. While no formal benchmarks for MINDDS-Connect are available, metadata queries currently return rapidly considering the minimal data transfer. Updates to the platform involve low-overhead Docker image pulls, with no downtime across other centers.

### Moving forward in genetics and mental health research

For patients with NDDs, a correct and early genetic diagnosis is crucial; early detection avoids a ‘diagnostic odyssey’ and enables rapid intervention, which can improve the development [29]. Timely management of child development and determining recurrence risks are vital for families. However, despite continued efforts, currently, about 60% of patients remain without a precise genetic diagnosis, representing a significant problem for patients and their families [30, 31]. Through our collaborative platform, MINDDS-Connect, we aim to address some current issues and enhance the diagnostic yield.

By addressing the challenges of data sharing, privacy, and standardization, MINDDS-Connect represents an advancement in mental health research. MINDDS-Connect emphasizes data quality and consistency. The platform incorporates rigorous data curation and quality control measures to ensure the reliability and accuracy of the shared data. This is crucial for drawing meaningful conclusions from the data analysis. Meta-analyses and large-scale studies have the potential to drive improved therapeutic strategies, identify new drug targets, and inform health policies, contributing to the advancement of Precision Medicine in mental health.

Additionally, federated approaches have become a focus point in biomedical research over the past years [5]. For example, the European Autism Genomics Registry (EAGER) plans to use federated data-integration mechanisms to integrate genetic, phenotypic, and clinical data across multiple sites in Europe. Leveraging initiatives like the Autism Sharing Initiative (ASI) and ELIXIR, EAGER aims to facilitate decentralized access to autism-related datasets while ensuring data privacy and regulatory compliance [32].

With federated approaches gaining traction in biomedical sciences, platform’s design and model can benefit other areas of research. By making MINDDS-Connect open source, other centers worldwide will be able to access the platform, adapt it to their needs, and build additional functionalities on top of it. MINDDS-Connect is developed as part of the broader WiNGS ecosystem supported by ELIXIR infrastructure (https://elixir-europe.org/). The initiative originated from a COST Action network and is now sustained by a the community of researchers and institutions that significantly contribute to NDD research. As more centers join and contribute data, the platform’s value will grow, fostering an increasingly collaborative research environment. A collaborative approach is essential for addressing complex challenges in mental health disorders, ultimately benefitting the patient’s well-being.

In conclusion, MINDDS-Connect addresses key challenges in NDD research with a secure, standardized platform for data sharing and analysis, with the potential to transform mental-health research, diagnosis, treatment, and policy.

## Supplementary information

Supplementary file 1

Supplementary file 2

Supplementary file 3

Supplementary file 4

Supplementary file 5

Supplementary file 6

## Data Availability

GitHub: https://github.com/wings-public/mindds-connect. Login page: https://wings.esat.kuleuven.be/Account/Login. Test account: minddsconnect_test@vermeeschlab.be pwd: 1Test-Account2 (no sample upload allowed).
