# Supplementary material for: MINDDS-connect: a federated data platform integrating biobanks for meta cohort building and analysis
Source: Eur J Hum Genet. 2025 Aug 20;33(11):1539–46. doi: 10.1038/s41431-025-01927-5 (PMC12583608; doi:10.1038/s41431-025-01927-5)
Supplement: Supplementary file 3 [file 41431_2025_1927_MOESM3_ESM.docx]

**Shared Samples**While similar to “Catalog,” the "Shared Samples" only displays samples explicitly shared with the current user, this allows for sharing of specific samples exclusively with certain users

**Activate User**:
This section is accessible only to Local Administrators (LAs) and allows them to link newly registered users to Principal Investigators (PIs) and register new PIs in the system.

**Dataset**:
Accessible to both LAs and PIs, this section provides tools for managing datasets, including viewing, adding, or updating data relevant to research activities.

**Group**:
This section enables LAs and PIs to manage groups, facilitating organization and collaboration within the research network by linking datasets and users.

**PI**:
Accessible to LAs and PIs, this section provides a centralized view of information related to a PI, such as linked users, associated datasets, and group memberships.
