# Supplementary material for: MINDDS-connect: a federated data platform integrating biobanks for meta cohort building and analysis
Source: Eur J Hum Genet. 2025 Aug 20;33(11):1539–46. doi: 10.1038/s41431-025-01927-5 (PMC12583608; doi:10.1038/s41431-025-01927-5)

User Guide

1. Catalog View


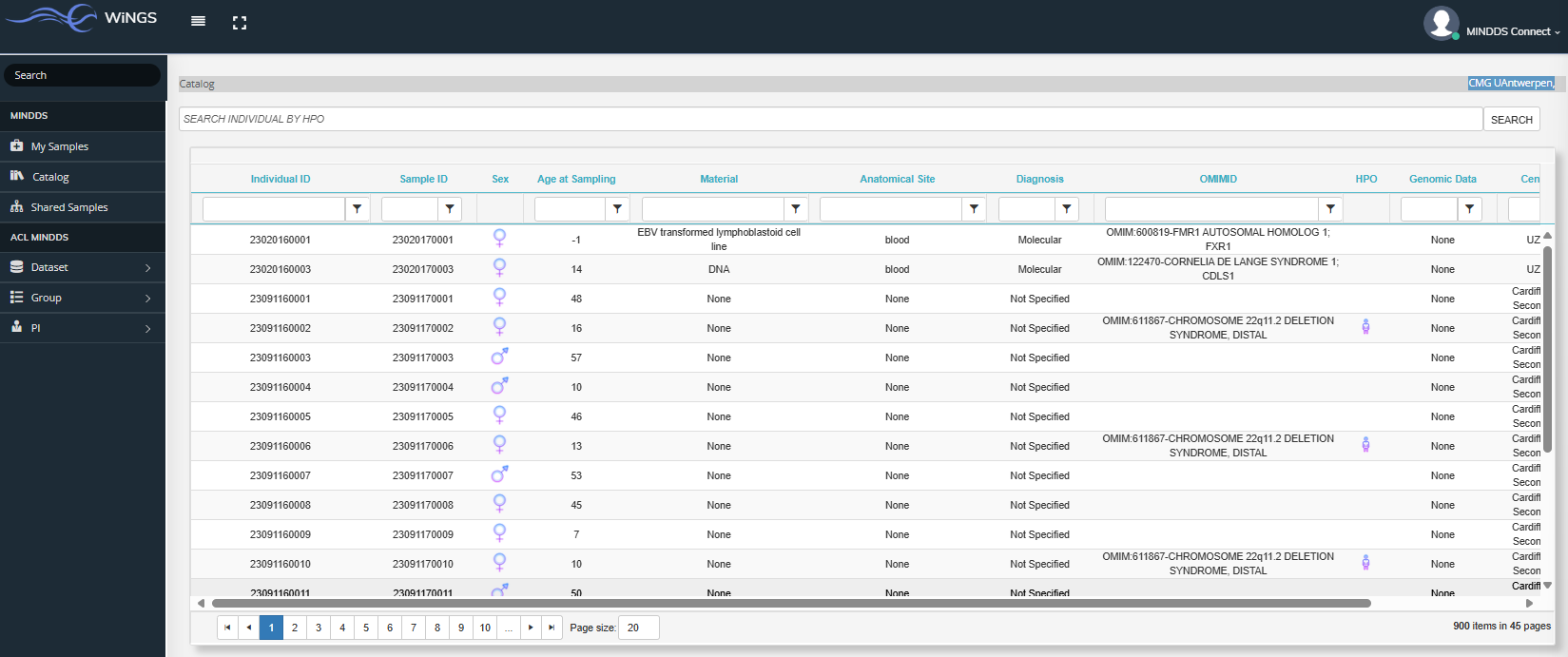


1. SELECT OMIM and *Apply*
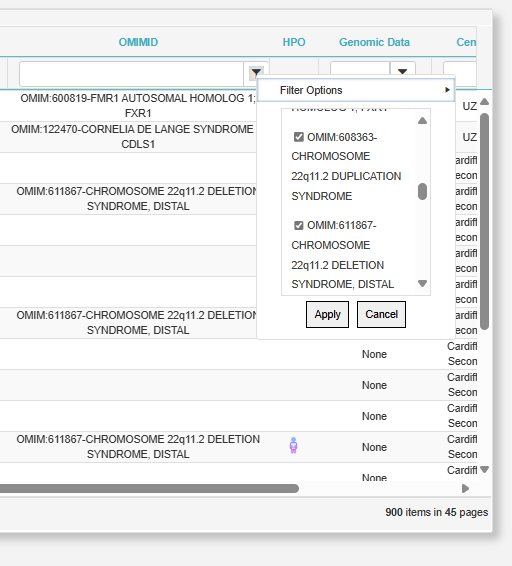

2. HPO Search
   1. Type into HPO search bar HPOs of interest and press *Search*


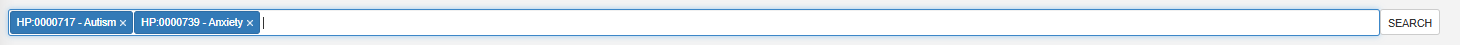


- 1. This will return a list of individuals with the desired HPO, click *Filter Table with Individuals*


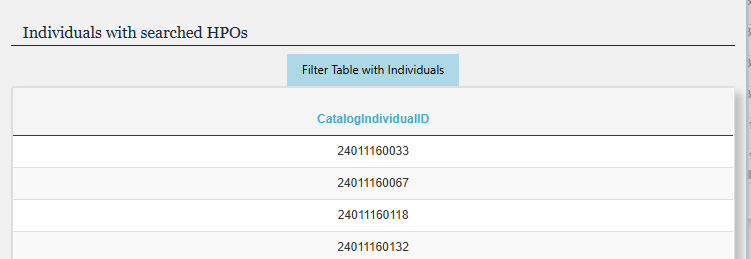


1. Result – Individuals with Selected OMIM and searched HPOs


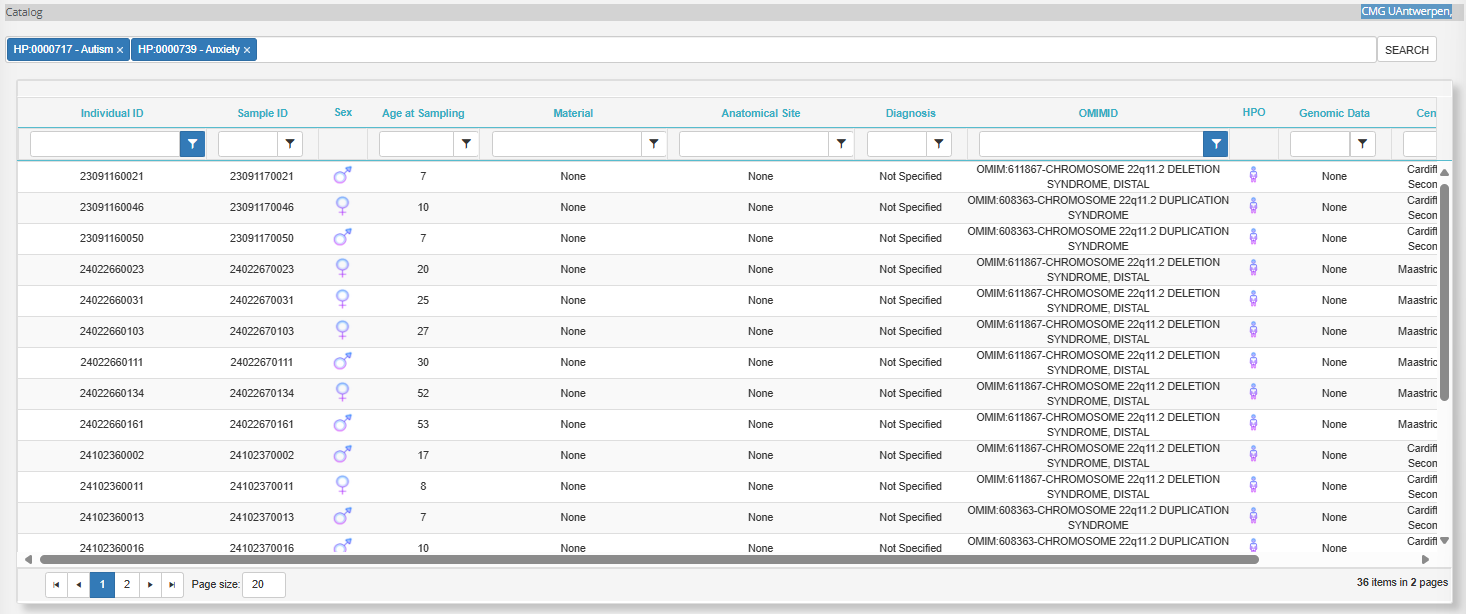


1. Sex based filter – select sex for individuals and apply


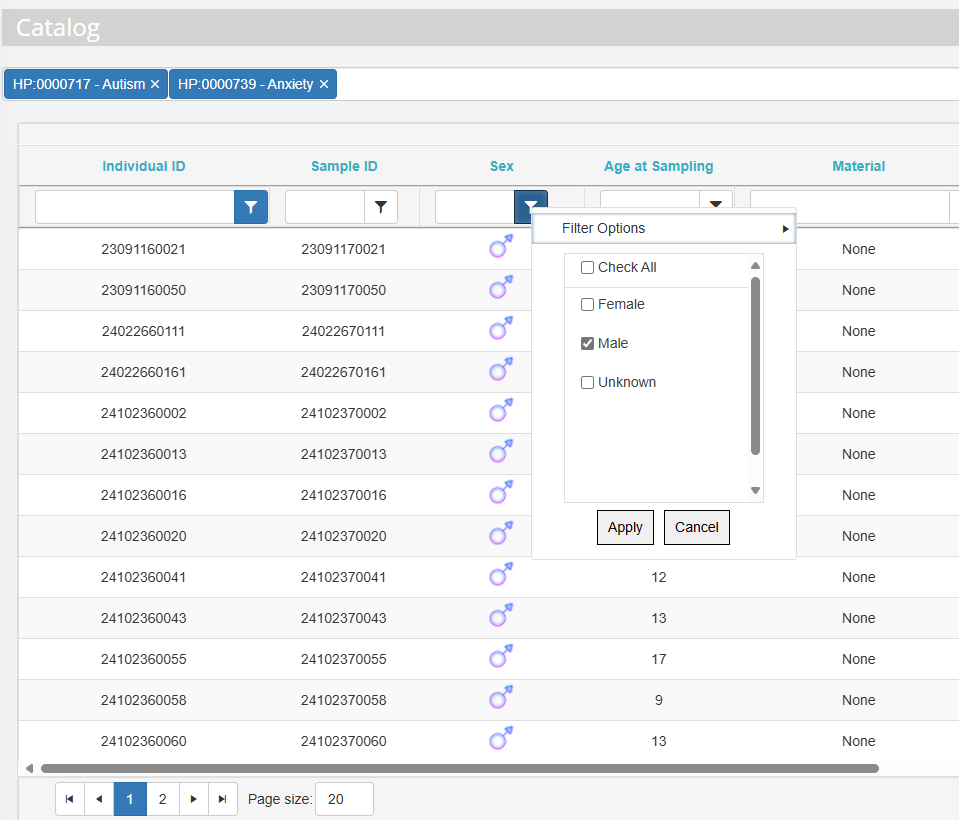

Supplement: Supplementary file 5 [file 41431_2025_1927_MOESM5_ESM.docx]
