# Supplementary material for: MINDDS-connect: a federated data platform integrating biobanks for meta cohort building and analysis
Source: Eur J Hum Genet. 2025 Aug 20;33(11):1539–46. doi: 10.1038/s41431-025-01927-5 (PMC12583608; doi:10.1038/s41431-025-01927-5)
Supplement: Supplementary file 6 [file 41431_2025_1927_MOESM6_ESM.docx]

**Federated Architecture**

WiNGS is built on a federated architecture that separates central coordination from on-premise computation. The **WiNGS Central Infrastructure (WiNGS-CI)** serves as the access point for participating centers. It authenticates users, enforces access control policies, and routes every request to the appropriate client instance. A C#/.NET-based web interface provides user management and administrative functions. Data as user accounts, principal investigators (PIs), datasets, and system-generated identifiers are stored in a Microsoft SQL Server database.

Each participating institution runs one or more **WiNGS-Clients** behind its own firewall. These client installations communicate exclusively with the WiNGS-CI over SSL, using JSON Web Tokens (JWTs) to ensure that only authenticated and authorized requests are processed. All data storage and compute services—variant storage, annotation modules, and local query handling are encapsulated within Docker containers on the client side, guaranteeing the safety of sensitive data

**Containerization**

All core WiNGS-Client components are distributed as Docker images, simplifying deployment and ensuring consistency across centers. This includes the **Client-API**, MongoDB instance. By leveraging Docker, upgrades to the platform reduce to pulling updated containers.

The **Node.js Client-API** is the gateway to on-premise data. It validates incoming JWTs issued by the WiNGS-CI, applies local ACL rules, translates queries into MongoDB operations, and returns results as JSON payloads. Security is enforced via SSL/TLS on all endpoints and JWT verification.

Underpinning the Client-API, **MongoDB** provides a flexible, schema-less data store ideally suited to the evolving needs of genomic and phenotypic metadata.

**Central Access Control (WiNGS-ACL)**

Access to data across the WiNGS ecosystem is governed by a hierarchical **WiNGS-ACL** managed centrally within the WiNGS-CI. At the top level, each client center appoints a **Local Administrator (LA)**, responsible for registering **Principal Investigators (PIs)** and issuing user accounts. PIs serve as custodians of their data, and **Users** inherit access by being linked to one or more PIs or by membership in user-defined groups that may span multiple centers.

Data itself is organized into **Individuals**, each linked to one or more **Samples**. For collaborative projects, PIs can define custom “datasets”, subsets of samples aggregated for a specific study, and share these cohorts with users or groups across centers. All ACL decisions are authored in the WiNGS-CI and enforced locally by the Client-API, ensuring that every request respects both user privileges and data-sharing agreements.
